# Comparison of Surgical Times Between Manual and Robot-Assisted Epiretinal Membrane Peeling

**DOI:** 10.1167/tvst.13.8.27

**Published:** 2024-08-14

**Authors:** Alexander Eberle, Ferhat Turgut, Gábor Márk Somfai, Amr Saad, Marc D. de Smet, Pascal W. Hasler, Florian M. Heussen, Matthias D. Becker

**Affiliations:** 1 Department of Ophthalmology, Stadtspital Zürich, Zurich, Switzerland; 2 Werner H. Spross Foundation for the Advancement of Research and Teaching in Ophthalmology, Zurich, Switzerland; 3 Department of Ophthalmology, Semmelweis University, Budapest, Hungary; 4 Gutblick Research, Pfäffikon, Switzerland; 5 Helvetia Retina Associates, Lausanne, Switzerland; 6 New York Eye and Ear Infirmary of Mt. Sinai, Icahn School of Medicine, New York, NY, USA; 7 Department of Ophthalmology, University of Basel, Basel, Switzerland; 8 Department of Ophthalmology, Bern University Hospital, Bern, Switzerland; 9 Department of Ophthalmology, University of Heidelberg, Heidelberg, Germany

**Keywords:** epiretinal membrane (ERM) peeling, robot assisted (RA), robotic surgery, operative times

## Abstract

**Purpose:**

Epiretinal membranes (ERM) pose a common challenge in vitreoretinal pathology, often causing vision impairment in older adults. The Preceyes Surgical System (PSS) supports the surgical removal of ERM through robot-assisted membrane peeling (RA-MP). This study compares surgical times and iatrogenic hemorrhages between manual membrane peeling (MMP) and RA-MP using PSS.

**Methods:**

Nine patients underwent RA-MP with PSS, whereas 16 patients (18 eyes) underwent MMP for comparative analysis. Surgical durations were categorized into RA-MP, manual forceps utilization in PSS surgeries (mRA-MP), and traditional MMP. Cumulative manual manipulation duration (cMMP), instrument grasps, and intraoperative hemorrhages were statistically analyzed using the Mann-Whitney *U* test.

**Results:**

RA-MP showed significantly longer peeling times compared to MMP (*P* < 0.001). Flap initiation grasps were similar between methods (*P* = 0.86), RA-MP demonstrated a significant reduction in peeling grasps (*P* = 0.01) and mean grasps per minute (*P* < 0.001). Although RA-MP resulted in fewer hemorrhages, the difference did not reach statistical significance relative to MMP (*P* = 0.08).

**Discussion:**

Although RA-MP tended to extend surgical time, it offered advantages in reducing tissue trauma and intraoperative hemorrhages. Further research is needed to explore the learning curve for novice surgeons and evaluate the safety profile of RA-MP.

**Translational Relevance:**

RA-MP may offer potential advantages over manual surgery, particularly in terms of reduced tissue trauma and intraoperative hemorrhages. Despite its longer duration compared with manual techniques, RA-MP may lead to fewer grasping maneuvers and lower rates of hemorrhages, thereby enhancing the safety and precision of vitreoretinal surgeries.

## Introduction

An epiretinal membrane (ERM) is a frequent vitreoretinal complication in which a thin layer of fibrous tissue grows on the inner surface of the retina.^1^ ERM typically affects older adults and can produce a variety of visual symptoms, such as metamorphopsia and decreased visual acuity, affecting near activities.^2^ ERM may develop due to intraocular inflammation, following retinal laser photocoagulation, cryocoagulation, pars plana vitrectomy, or be idiopathic in nature. The ERM may remain stable, without causing any visual symptoms, or can progress to macular pseudoholes and lead to permanent vision loss, severely impacting the quality of life.^2^ Despite extensive research, the pathogenesis of ERM is not well understood; however, at least two mechanisms are repeatedly mentioned: age-related changes of the vitreous body associated with vitreoschisis,^3^ and glial cell proliferation on the vitreoretinal interface. The prevalence of ERM rises dramatically with age; a recent meta-analysis found an overall age-standardized ERM prevalence of around 9%, with considerable geographic variability.^4^

Surgical removal of visually significant ERMs is the only option to restore vision. Optical coherence tomography (OCT) is typically used to visualize and guide the treatment of ERM.^5^ The current standard of care for ERM is the surgical removal by membrane peeling (MP), combined with a pars plana vitrectomy (PPV), which was pioneered by German American ophthalmologist Dr. Robert Machemer in the 1970s.^6^ MP has a high success rate (improvement in vision in 75% to 85%) and a low incidence of complications, with the majority of patients experiencing considerable improvement in visual acuity and quality of life.^7^ The potential early and late complications of MP include the possibility of ERM recurrence, increased intraocular pressure, iatrogenic retinal trauma, intraocular infection, hemorrhage, retinal detachment, microcystic macular edema, and cataract formation.^8^^–^^14^

Medicine has benefited significantly from developments in computer sciences and machine learning. There is also a growing interest in the use of robotic systems in surgery, and with improvements in miniaturization, it is now possible to consider the same in vitreoretinal surgery. The Preceyes Surgical System (PSS) was developed for use in ophthalmic surgery, including MP.^15^ We previously used this robotic system for MP and recorded the times necessary for the entire surgical procedure. Although robotic systems hold great promise for improving ERM surgery, they also have drawbacks. One concern is the increased time required to set up and use the PSS, leading to prolonged surgical times.^16^ However, in the performance of high-precision tasks, even in manual surgery, an expenditure of time is expected. A benefit of increased precision should be lesser trauma, such as retinal hemorrhages, and it should require less “grasps” of the ERM. The aim of this study was to compare surgical times for manually performed MP (MMP) and robot-assisted MP (RA-MP) using the PSS and correlating these with the number of intraoperative grasps performed by the surgeon, as well as any induced trauma.

## Patients and Methods

This retrospective monocentric study included 9 eyes of patients who received RA-MP and 18 eyes of 16 patients who underwent MMP at the Department of Ophthalmology, Stadtspital Zürich, Switzerland, between January 2021 and December 2022. The study was approved by the Zurich Cantonal Ethics Commission (BASEC-Nr. 2023-01867).

All patients provided informed written consent for their participation in this research.

**Figure 1.**
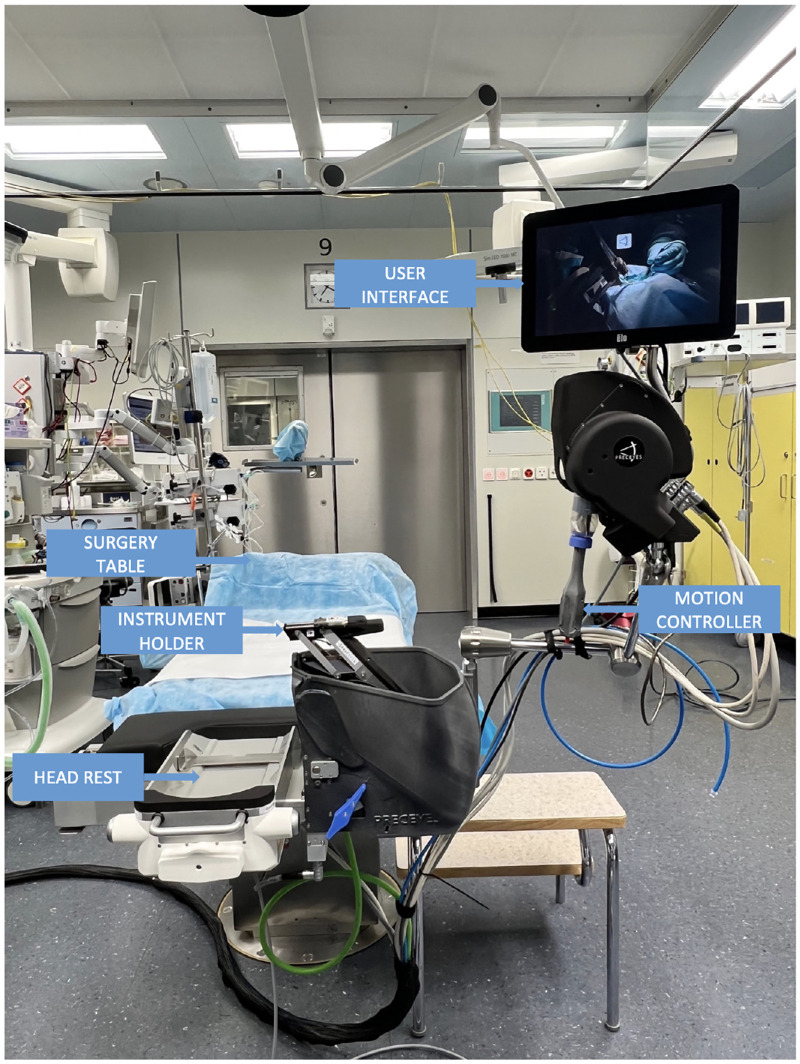
An overview of the Preceyes Surgical System (PSS). The system uses a telemanipulation mechanism, wherein the motion controller functions akin to a joystick, translating coarse movements into precise adjustments of the intraocular device. This tool is attached to the instrument holder, gaining access to the eye through a trocar. Modules are selected through a touchscreen interface, while the foot switch enables pneumatic opening and closing of the forceps.

The setup can be seen in Figure 1, whereas the detailed methodology of PSS usage, including the training of the surgeons and PSS setup, are described in detail in our previous paper.^16^ During the PSS procedure, in case a switch to manual surgery was necessary, the documentation was continued as a manual procedure.

Parameters, including the number of grasps (for flap initiation and peeling), instances of hemorrhages, and surgical duration, including adverse events, were assessed by a single observer who conducted a retrospective analysis of all surgical videos. The recordings, obtained through the ARTEVO surgical microscope with a frame rate of 50 to 60 frames per second (Carl Zeiss Meditec, Jena, Germany), featured precise time stamps. The start and end points for evaluating the surgical duration were determined based on the visibility of the forceps (23-gauge end-gripping forceps; Optico Ltd., Letchworth Garden City, United Kingdom) through the microscope. In cases where the forceps temporarily vanished from view for less than 3 seconds (quick wiping), the timer continued running. However, if the forceps remained out of sight for more than 3 seconds, the timer was paused. An initiation grasp was defined as a forceps maneuver that did not involve peeling but instead entailed visible manipulation of the ERM/inner limiting membrane (ILM) through pulling or lifting. Grasps where the forceps made no visible contact with the ERM/ILM (empty grasps) were not classified as valid grasps. A peeling grasp was characterized by forceps movements that resulted in the visible separation of a portion of the ERM. Hemorrhage was defined as the occurrence of pinpoint or blot-like bleeding, either following peeling or after an initiation grasp.

Data were recorded for RA-MP, the manual usage of forceps during surgeries with PSS (mRA-MP), and manually performed MMP. The timing for using either the manual forceps or the PSS started when the forceps became visible inside the eye and ended as soon as the forceps was removed from the eye's interior. The data for the manual manipulations during surgeries with or without the PSS, the number of grasps, and hemorrhages were compared with the RA-MP group. Statistical analysis was performed using a Mann-Whitney *U* test to compare the parameters between the two groups.

## Results

The descriptive data of the surgical times, flap initiation grasps, peeling grasps, grasps per minute, and hemorrhages can be seen in the Table. In PSS cases where a switch to manual manipulation of the forceps was necessary, the data were recorded separately and used for the calculations.

**Table.** Surgical Metrics for Robot-Assisted and Manual Membrane Peeling (RA-MP, Respectively MMP)

Surgical Metrics for Robot-Assisted and Manual Membrane Peeling							
	*N* (Male/Female)	Age in Years Mean (95% CI)	Time in Seconds Mean ± SD (95% CI)	Flap Initiation GraspsMean ± SD (95% CI)	Peeling GraspsMean ± SD (95% CI)	Grasps Per MinuteMean ± SD (95% CI)	Hemorrhages Per Grasp Mean
RA-MP	9 (4/5)	67 (57–77)	1189 ± 378	7.1 ± 5.3	11.9 ± 10.7	0.9 ± 0.4	0.6 (0.045)
			(942–1436)	(3.6–10.6)	(4.9–18.9)	(0.6–1.2)	
mRA-MP	7 (4/3)	74 (71–77)	272 ± 121	11 ± 7.1	14.7 ± 8.5	5.9 ± 1.9	1.7 (0.076)
			(183–362)	(5.7–16.3)	(8.4–21.0)	(4.5–7.2)	
MMP	18 (8/10)	77.6 (67–73)	256 ± 97	6 ± 6.1	28.4 ± 13.6	8.4 ± 3.2	1.7 (0.064)
			(210–301)	(3.2–8.8)	(22.2–34.7)	(6.9–9.9)	
PSS (= RA-MP ∪ mRA-MP)	9 (4/5)	67 (57–77)	788 ± 550	8.8 ± 6.3	13.1 ± 9.6	3.1 ± 2.8	1.1 (0.058)
			(518–1057)	(5.7–11.9)	(8.4–17.8)	(1.7–4.5)	
cMMP (Σ of MMP + mRA-MP)	25 (12/13)	76.6 (68–74)	261 ± 102	7.4 ± 6.7	24.6 ± 13.7	7.7 ± 3.1	1.7 (0.067)
			(220–301)	(4.8–10.0)	(19.2–30.0)	(6.5–8.9)	

95% CI, 95% confidence interval; cMMP, cumulative manual membrane peeling; MMP, manual membrane peeling; mRA-MP, additional manual manipulation during PSS surgeries; PSS, PrecEyes Surgical System; RA-MP, robot-assisted membrane peeling.

The duration of the peeling process was approximately fourfold longer for RA-MP compared to MMP (*P* < 0.001; see the Table). No statistically significant difference was found for flap initiation grasps (*P* = 0.86), whereas there were significantly fewer peeling grasps performed in the RA-MP group (*P* = 0.01). The mean grasps per minute was significantly lower in RA-MP cases than that of the cumulative manual manipulation duration (cMMP, *P* < 0.001), which is consistent with the longer manipulation time. Regarding the hemorrhages during or after manipulation, RA-MP had fewer hemorrhages, but this did not reach statistical significance as compared to the manual group (*P* = 0.08) and the hemorrhages per grasp were also not significantly different between the two groups (*P* = 0.32). Considering the grasps per minute difference, there were significantly fewer hemorrhages per minute in RA-MP (mean cMMP = 0.49, mean RA-MP = 0.032, *P* = 0.016).

**Figure 2.**
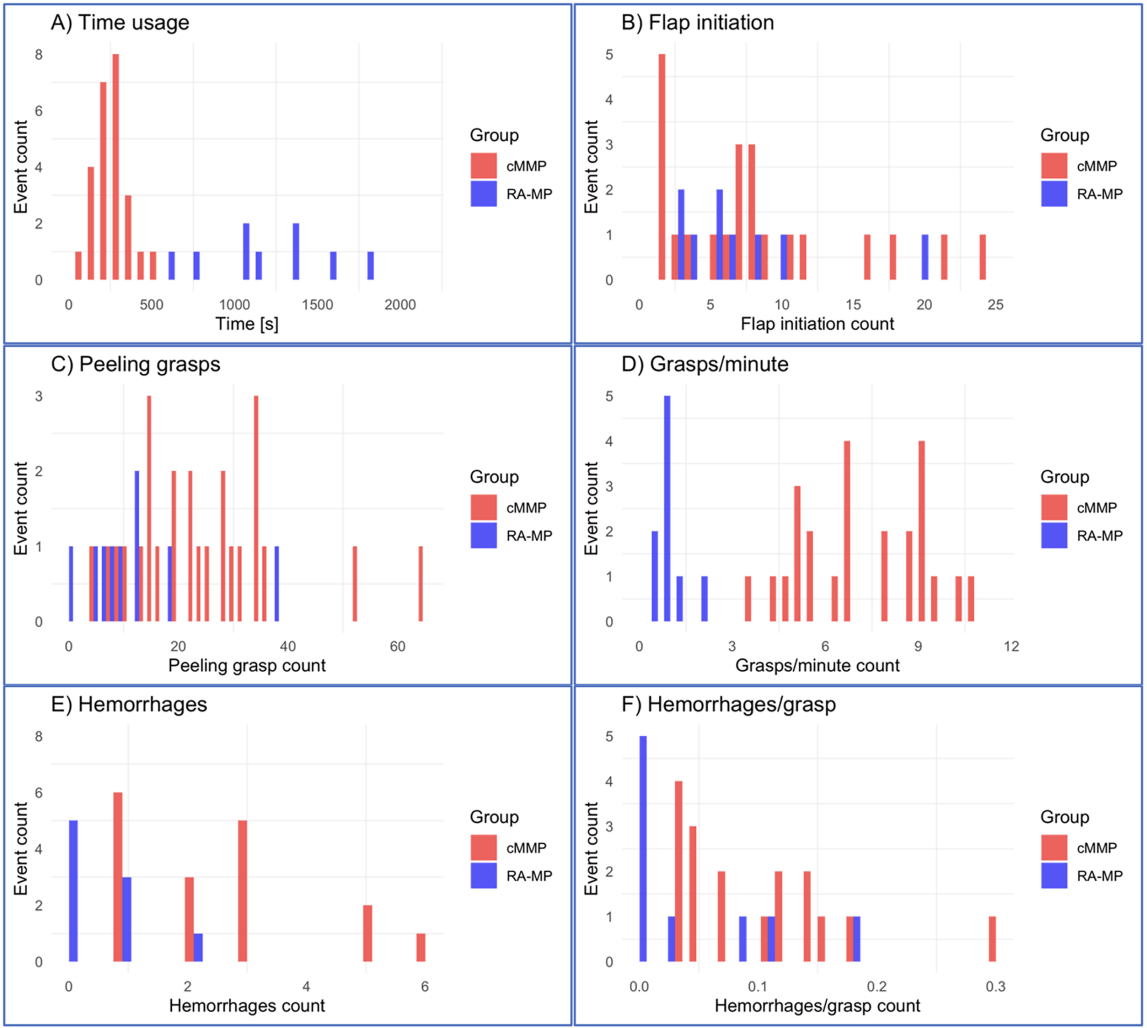
(**A–F**) Histograms for the manual (*red*) and the PSS (*blue*) groups. (**A**) Surgical time distribution. (**B**) Flap initiation grasps. (**C**) Peeling grasps. (**D**) Grasps per minute. (**E**) Hemorrhages. (**F**) Hemorrhages per grasp. cMMP, values for the cumulative manual membrane peeling (summation of MMP and mRA-MP); RA-MP, values for the robot-assisted surgeries.

To better visualize the distribution of the data, we opted to use histograms as a visual aid, with the x-axis representing the parameter in question and the y-axis indicating its frequency of occurrence. Figures 2A through 2F present the distributions of the parameters studied. Patterns within the context of statistically significant comparisons (see Figs. 2A, 2C, 2D) emerge, which exhibit pronounced bimodal tendencies in the case of Figures 2A and 2D, and notably distinct density distributions in the case of Figure 2C.

As mentioned above, it can be observed that RA-MP was significantly more prolonged in its application (*P* < 0.001; see Fig. 2A). A significant difference in terms of peeling grasps between the two groups can be illustrated (*P* = 0.01; see Fig. 2C). The last significantly distinct parameter is the grasps per minute (see Fig. 2D), which clearly demonstrates separate value ranges for each group (*P* < 0.001).

Conversely, for comparisons deemed statistically nonsignificant (see Figs. 2B, 2E, 2F), a conspicuous absence of discernible trends is evident. A similar distribution regarding flap initiation has been displayed in Figure 2B, which leads to no significant differences between cMMP and RA-MP (*P* = 0.86). Total hemorrhages and hemorrhages per grasp similarly exhibit a very similar distribution for both groups, indicating no significant differences (*P* = 0.08 and *P* = 0.32; see Figs. 2E, 2F).

## Discussion

In our study, we conducted a comparative analysis of the surgical durations and procedural aspects of cMMP to RA-MP. The results revealed that although RA-MP had longer durations, there were fewer peeling grasps necessary. This outcome suggests that the robotic system may offer improved control and stability during surgical maneuvers, potentially reducing the risk of hemorrhages. An alternative interpretation could be that the surgeons approached the robotic technique with greater caution due to their unfamiliarity with it and the fact that pneumatically initiated opening and closing of the microsurgical forceps with the foot pedal is cumbersome compared to the manual technique. This finding raises the question of how the outcomes would differ in novice surgeons, warranting exploration in future research endeavors.

In other surgical disciplines, studies have shown that robotic systems can offer increased precision, reduce tissue trauma, improve surgical outcomes, and, in some cases, even financial advantages due to shorter hospital stays and fewer follow-up interventions.^17^^–^^19^ However, precision tasks require time, both manually and robotically, identifying the right tradeoff between time and precision has so far been difficult with all medical robotic systems.^20^

The increased surgical time associated with the robotic system is largely attributable to the technology's setup and integration into the surgical workflow, which is in line with previously reported concerns.^18^^,^^21^^,^^22^ However, it is important to note that the use of the PSS is still at an early stage, and with further advancements and refinements, the setup time could be reduced, leading to more efficient robot-assisted surgeries.

Moreover, the limited experience of the two surgeons in our study, having performed only four and five operations using the robotic system, raises considerations regarding the learning curve and resulting outcomes with the platform. Exploring the impact of surgical time and tissue damage in the case of novice surgeons could give a better insight into the learning curve and the acquisition of robotic surgical skills compared to manual techniques. A previous study by Jacobsen et al., using a simulator, found no significant difference in the learning curves between RA-MP and MMP.^23^

When considering the occurrence of hemorrhages per grasp, no significant differences were observed between the manual group and the robotic group. This indicates that the frequency of hemorrhages may not be directly correlated with the number of grasps performed during the procedure. Despite this lack of significant difference, our findings hold substantial implications for the future adoption of RA-MP. Surgeons with extensive experience in manual operations but limited exposure to robot-assisted procedures displayed a comparable incidence of bleeding events per grasp. This suggests a promising avenue for the future, particularly for novice surgeons who have received specialized training in robotic surgical techniques. Additionally, factors such as the force applied during grasping or the inherent characteristics of the ERM itself may also contribute to the occurrence of hemorrhages, warranting further investigation.

It is also imperative to delve into the intricate nature of hemorrhages, which can manifest in two distinct forms. Immediate occurrences may likely result from direct capillary or venular damage, whereas delayed instances suggest oozing from the peel site. Notably, the latter phenomenon appears intricately linked to the extraction of the neuromuscular complex from the retina, accompanied by adherent Muller cells to the undersurface of the membrane. An interesting correlation may emerge when assessing the nerve fiber layer loss at 6 months, potentially corresponding with the location of the hemorrhages and potentially worsening with delayed onset. This prompts consideration for the collection of internal limiting membranes from intraoperative peels, with electron microscopy offering insight into the presence of the neuromuscular complex.

It is important to underline the limitations of our study, including the small sample size and the retrospective analysis of surgical videos. We believe that due to the limited observations in the field, our initial experiences may be of importance for the planning of future studies and may also serve as a reference for the future development and implementation of robotic systems in vitreoretinal surgery. Additionally, the limited experience level of the surgeons with the robotic system may have affected the outcomes; also in addition, we did not include the assessment of structural and functional outcomes in the paper due to our main focus on the surgical technique itself. With larger surgical volumes by the PSS, a more realistic view can be obtained, which is why we are planning a larger, prospective study in the future with more defined anatomic and functional outcomes.

## Conclusions

Our small, retrospective study suggests that although RA-MP may take a longer time than manual techniques, it offers potential advantages in terms of fewer grasping maneuvers and the possibility of lower rates of intraoperative hemorrhages, thus improving the safety and precision of vitreoretinal surgeries. Further studies with larger sample sizes and prospective designs are necessary to validate and expand upon our findings.
